# Evaluation of the X-ray/EUV Nanolithography Facility at AS through wavefront propagation simulations

**DOI:** 10.1107/S1600577524002534

**Published:** 2024-04-17

**Authors:** Jerome B. M. Knappett, Blair Haydon, Bruce C. C. Cowie, Cameron M. Kewish, Grant A. van Riessen

**Affiliations:** a Department of Mathematical and Physical Sciences, School of Computing, Engineering and Mathematical Sciences, La Trobe University, Bundoora, Victoria 3086, Australia; b Australian Synchrotron, Australian Nuclear Science and Technology Organisation (ANSTO), Clayton, Victoria 3168, Australia; RIKEN SPring-8 Center, Japan

**Keywords:** extreme ultraviolet lithography, soft X-ray lithography, interference lithography, wavefront propagation, synchrotron beamline

## Abstract

Synchrotron light sources can provide the required spatial coherence, stability and control to support the development of advanced lithography at the extreme ultraviolet and soft X-ray wavelengths that are relevant to current and future fabricating technologies. Here an evaluation of the optical performance of the soft X-ray (SXR) beamline of the Australian Synchrotron (AS) and its suitability for developing interference lithography using radiation in the 91.8 eV (13.5 nm) to 300 eV (4.13 nm) range are presented. A comprehensive physical optics model of the APPLE-II undulator source and SXR beamline was constructed to simulate the properties of the illumination at the proposed location of a photomask, as a function of photon energy, collimation and monochromator parameters. The model is validated using a combination of experimental measurements of the photon intensity distribution of the undulator harmonics. It is shown that the undulator harmonics intensity ratio can be accurately measured using an imaging detector and controlled using beamline optics. Finally, the photomask geometric constraints and achievable performance for the limiting case of fully spatially coherent illumination are evaluated.

## Abstract 2

The optical performance and suitability for EUV interference lithography of the soft X-ray beamline of the Australian Synchrotron is evaluated through comparisons of partially coherent simulation and experimental measurements.

## 1. Introduction

As of 2019, the semiconductor device manufacturing industry has adopted lithography technology utilizing extreme ultraviolet (EUV) radiation of wavelength λ = 13.5 nm, corresponding to a photon energy of 91.8 eV, for high-volume manufacturing (Fomenkov, 2019 ▸). To keep up with the demands of device scaling predicted by Moore’s law, a future transition to 6.7 nm wavelength (185 eV) sources is anticipated. The availability of laser-pulsed plasma light sources (Otsuka *et al.*, 2012 ▸) and multilayer optics (Uzoma *et al.*, 2021 ▸) at this wavelength make it a particularly promising candidate for future lithography. A shift to shorter wavelengths brings with it significant challenges, including an increased significance of stochastic effects for higher resolution patterning (De Bisschop, 2017 ▸), and a need for understanding the wavelength-dependent effects of mask defects (Goldberg & Mochi, 2010 ▸). Interference lithography (IL) using synchrotron radiation has recently emerged as a powerful tool for understanding the challenges for future lithographic processes, including photoresist performance from EUV (Mojarad *et al.*, 2015*a* ▸) to soft X-ray (SXR) wavelengths (2.5 nm) (Mojarad *et al.*, 2021 ▸). Synchrotron sources are ideal for the development of lithographic technology as they allow for the control of properties such as flux, coherence and polarization, and can cover the entire energy range from EUV to SXR. Partially coherent wavefront propagation simulation can provide critical insight into the design of optical systems and EUV/SXR-IL process development using existing synchrotron radiation sources.

For high-resolution patterning using IL, it is critical to achieve high contrast in the aerial image formed where beams diffracted from two or more gratings interfere. This requires that light sources used in IL provide high intensity, spatial coherence and stability (Mojarad *et al.*, 2015*b* ▸). The necessary spatial coherence length to ensure high-contrast aerial images is determined by the size of the desired exposure area (Solak *et al.*, 2003 ▸), which is, in turn, defined by the maximum separation between points in a set of two or more radially arranged gratings. Fully coherent illuminaton of the grating set typically requires a coherence length three times the linear dimensions of the desired exposure area (Solak *et al.*, 2002 ▸). Typical grating masks used for EUV-IL require a lateral coherence length at the mask plane of between 150 µm and 1.2 mm (Ekinci *et al.*, 2014 ▸; Meng *et al.*, 2021 ▸), although small area patterning using EUV-IL has been reported using a coherence length of just 43.2 µm (Sahoo *et al.*, 2023 ▸).

When evaluating the performance of a light source for interference lithography, the relevant quantity that determines throughput is the exposure time required to provide the radiation dose-on-wafer *D*_w_(λ) to transfer a pattern to a film of photosensitive resist with particular sensitivity at a particular wavelength λ. This, in turn, depends on the coherent intensity at the mask and mask efficiency. Practically, for studies of lithographic processes at high resolution, through­put is of lower priority than lithographic quality, and the exposure time determines the required mechanical stability between the mask and wafer.

Photoresist sensitivity is defined by the dose *D*_0_(λ) required to remove 50% of the resist thickness during development for a particular wavelength (Ekinci *et al.*, 2013 ▸; Mojarad *et al.*, 2015*a* ▸). Resist sensitivity generally decreases for shorter wavelengths, *i.e.*, *D*_0_ ∝ 1/λ. Combined with the requirement for higher doses for patterning thinner resists and smaller feature sizes, the scaling of source power requirements with industry device scaling targets is a major challenge (Van Schoot, 2021 ▸; Levinson, 2022 ▸). For the present work, it is convenient to define the dose-on-mask, *D*_m_, required to achieve a target value of *D*_0_, taking into account diffraction efficiency, η(λ), of the grating mask and the transmission of the substrate, *T*(λ), at the relevant wavelength, 

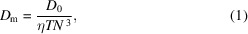

where *N* is the number of gratings contained in the mask and the power of three is due to the definition of the efficiency of an IL grating, η_IL_ = *N*η (Mojarad *et al.*, 2015*b* ▸), and periodicity in the aerial image intensity. The wavelength dependence has been suppressed for simplicity. While diffraction efficiencies of up to 28% have been reported for low-resolution IL gratings (Braig *et al.*, 2011 ▸), Wang *et al.* (2021 ▸) reported diffraction efficiencies for a bilayer of hydrogen silsesquioxane and spin-on-carbon of η > 6% for gratings with half-pitch down to 12 nm at EUV wavelengths.

Organic chemically amplified resists (CARs) have been used for performance comparisons of EUV and SXR, as they offer high-resolution patterning in both wavelength ranges (Mojarad *et al.*, 2013 ▸). Mojarad *et al.* (2015*a* ▸) reported CAR sensitivities of *D*_0_ (13.5 nm) = 11.6 mJ cm^−2^ and *D*_0_ (6.5 nm) = 33.2 mJ cm^−2^. The 2021 International Roadmap for Devices and Systems has predicted that *D*_0_ > 80 mJ cm^−2^ will be required for 10 nm half-pitch patterning (Levinson, 2022 ▸). Assuming a SiO_2_ substrate of 40 nm thickness and a four-grating mask with η = 6% efficiency, the corresponding minimum dose on mask for EUV-IL can be estimated to be *D*_m_ ≃ 11 mJ cm^−2^.

In this work, partially coherent wavefront propagation was used to simulate the complex wavefield at the mask plane of an EUV-IL instrument being developed at La Trobe University for one of two branches of the SXR beamline of the Australian Synchrotron (AS). Simulations were performed primarily at 90.44 eV and 184.76 eV photon energy and the relevant characteristics of the beam were compared with direct measurements of the intensity taken at the beamline with photon energy ranging from 90 eV to 300 eV. We demonstrate, by comparison with photoelectron spectroscopy measurements, that the ratio of undulator harmonic intensity can be measured using an imaging detector. We discuss how the higher harmonic intensity scales with decreasing photon energy and is affected by collimating slits also used to control spatial coherence. The beamline’s suitability for EUV-IL is evaluated with respect to the compromise between the grating area that can be coherently illuminated and the intensity of the illumination.

## 2. Theory and modelling

### 2.1. Interference lithography

Light incident on a grating diffracts at multiple angles, θ_*m*_, corresponding to orders, *m*, governed by the grating equation. For a monochromatic beam of wavelength λ, incident at angle θ_i_, on a grating with period, *p*_G_, the angle of diffraction of the *m*th order beam is (Hecht, 1987 ▸) 

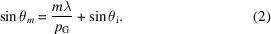

**Figure 1:**
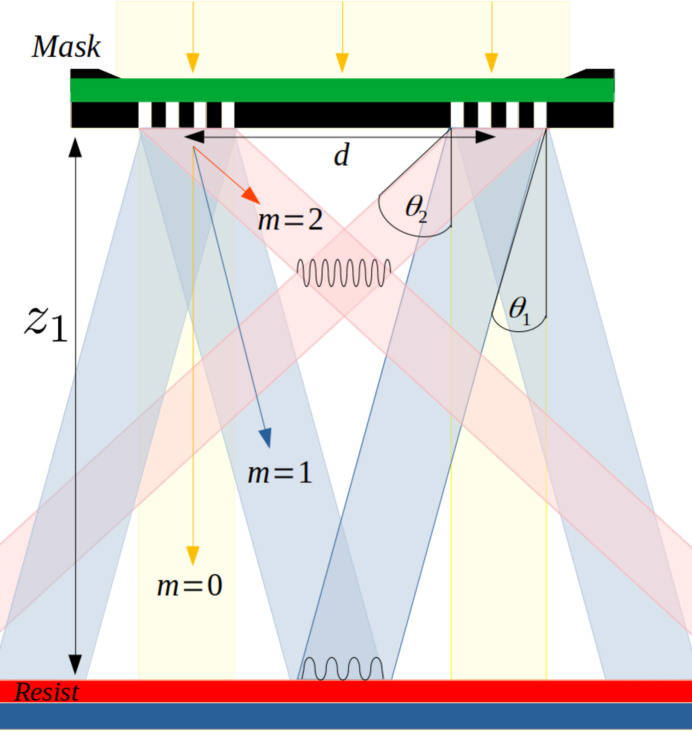
Schematic of an interference lithography setup using a binary grating mask. The first-order (*m* = ±1) beams form an aerial image at the image plane, a distance *z*_1_ from the mask, which is then transferred onto a photoresist. The zero-order (*m* = 0) and second-order (*m* = 2) diffracted beams are also shown.

As shown in Fig. 1 ▸, the *m*th-order beams from each grating will interfere at the aerial image plane at a distance *z*_*m*_ from the mask, given by

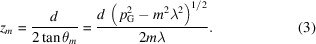

For the simplest IL setup, consisting of two plane waves intersecting at a 2θ_*m*_ angle, the aerial image will have a period *p* given by 

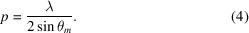

The standard measure of the resolution of an aerial image is the half-pitch, HP = *p*/2. For a grating illuminated at normal incidence, equations (2) and (4) can be combined to show that 

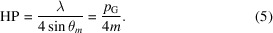

The ultimate resolution for a first-order aerial image is therefore *p*_G_/4 (Karim *et al.*, 2015 ▸). Equation (5) implies that sub-10 nm patterning by IL requires a grating mask with *p*_G_ < 40 nm, which can be readily fabricated using electron beam lithography (Vieu *et al.*, 2000 ▸). It also shows the achromatic nature of IL, as the right side of equation (5) is independent of λ.

### 2.2. Source model

**Figure 2:**
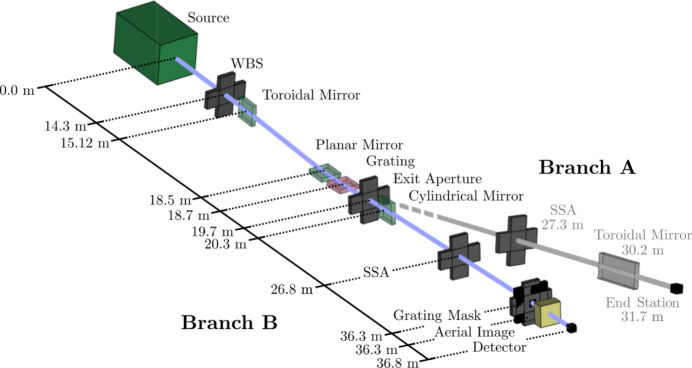
Schematic illustration of the SXR beamline. The distance of each element from the source is indicated (not to scale). Proposed IL optics and an imaging detector are included in branch B. Branch A was used to obtain measurements of the undulator harmonic intensity using photoelectron spectroscopy.

*Synchrotron Radiation Workshop* (*SRW*) (Chubar & Elleaume, 1998 ▸) was used to construct a model of branch B of the SXR beamline at the AS, shown in Fig. 2 ▸ (Knappett, 2021 ▸). A new model discussed in the present work includes the proposed EUV-IL endstation, permitting evaluation of the influence of all beamline parameters on the properties of the aerial image. *SRW* is commonly used to model SXR/EUV synchrotron beamlines and is capable of fully or partially coherent propagation (He *et al.*, 2020 ▸; Meng *et al.*, 2021 ▸).

#### 2.2.1. Storage ring and electron beam

The parameters used to model the electron beam and storage ring were obtained from Wootton *et al.* (2012 ▸, 2013 ▸) and are listed in Table 1 ▸.

#### 2.2.2. APPLE-II undulator

The SXR beamline utilizes an elliptically polarizing APPLE-II undulator source. This undulator was modelled as ideal, with magnetic fields defined by the undulator length, *L*, magnetic period, λ_u_, and number of periods, *N*_u_. The undulator parameters were obtained from Wootton *et al.* (2012 ▸, 2013 ▸) and are shown in Table 2 ▸.

### 2.3. Beamline model

A schematic of the optical elements in use for each branch of the SXR beamline is shown in Fig. 2 ▸. The key optical components include white-beam slits (WBS), a toroidal mirror, a planar grating monochromator (PGM) and a cylindrical mirror that can be rotated to direct the beam to branch A or B. The schematic shows the proposed location of IL optics in branch B, along with an existing EUV/SXR area detector. For typical operating conditions of branch B, the WBS are wide open (not touching the beam) and the bandwidth of the beam is defined by the PGM and secondary source aperture (SSA). The PGM disperses the monochromatic components of the incident beam at angles dependent on the photon energy as defined in equation (2), the beam is then focused at the SSA, the size of which defines the resolving power of the beam as (λ/dλ). All relevant parameters are listed in Table 3 ▸.

### 2.4. Partially coherent simulations

*SRW* implements fully spatially coherent wavefront propagation through the propagation of monochromatic radiation emitted by a single relativistic electron passing through an undulator. The wavefield is represented by the monochromatic, transverse components of the electric field *E*_⊥λ_ and paraxial propagation is performed through fast Fourier transforms (Chubar & Elleaume, 1998 ▸). Taking *x* and *y* as the horizontal and vertical directions, respectively, and *z* as the direction along the beam axis, the propagated wavefield, *E*_⊥λ_(*x*, *y*, *z*) from each wavefield component at *z* = 0, *E*_⊥λ_(*x*, *y*, *z* = 0), can be written as 

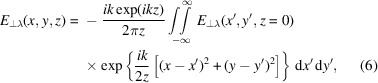

where *k* = 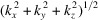 is the spatial frequency of the plane wave component over an area of phase space d*k*_*x*_d*k*_*y*_. In all simulations shown in this work, only a single monochromatic component is considered, where the spatial frequency *k* = 2π/λ, and a quadratic approximation to the exponential phase term in equation (6) (Chubar & Celestre, 2019 ▸) was used where appropriate.

Partially coherent monochromatic propagation is achieved by randomly sampling the phase space of the entire electron beam using the Monte Carlo method (Laundy *et al.*, 2014 ▸). The number of electrons needed to accurately represent the electron beam increases with the electron beam emittance (Chubar *et al.*, 2016 ▸). For all simulations shown in this work, 4000 electrons were sufficient to accurately model the monochromatic beam (Knappett, 2021 ▸). The electric field of each electron is then propagated coherently using equation (6) and the partially coherent electric field is calculated as the average of the electric field distributions from each electron, seeded over the phase space occupied by the beam (Chubar, 2014 ▸).

## 3. Simulation results

### 3.1. Beam profile

The beamline model was used to propagate a partially coherent wavefield from the source through to the grating mask plane at λ = 4.96, 6.7, 9.2 and 13.5 nm. The intensity was modelled from the complex electric field as *I*(*x*, *y*) = |*E*(*x*, *y*)|^2^, with the pixel size chosen such that it satisfies the Nyquist–Shannon sampling theorem to ensure satisfactory representation of phase (Shannon, 1949 ▸). The full width at half-maximum (FWHM) of the intensity profile at the mask plane was calculated for each wavelength.

### 3.2. Flux and coherence at the mask plane

The total flux and spatial coherence of the wavefield at the mask plane was evaluated after partially coherent propagation through the beamline model. The total flux, Φ, was calculated in units of photons per second per 0.1% bandwidth from the propagated complex electric field *E*(*x*, *y*) by 

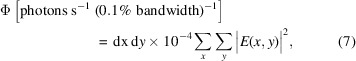

where d*x* and d*y* are the pixel size of *E* in *x* and *y*, respectively. The flux was corrected for the reflectivity of each mirror and efficiency of the grating used in the PGM, shown in Table 3 ▸. Since the reflection grating efficiency at EUV/SXR wavelengths is not accurately known, a constant efficiency of 10% was assumed for all simulations, with a third-order efficiency of 0.1%.

The spatial coherence length *l*_c_ at the mask plane was calculated by first computing the one-dimensional mutual intensity *J* using 

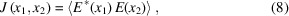

where *x*_1_ and *x*_2_ are different sets of points in the central horizontal axis (*x*, *y* = 0), so that when *x*_1_ = *x*_2_, *J* reduces to the horizontal intensity profile. The degree of coherence between any point along (*x*, *y* = 0) and the central point (*x* = 0, *y* = 0) was then calculated following Meng *et al.* (2021 ▸) using 

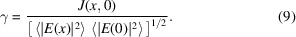

**Figure 3:**
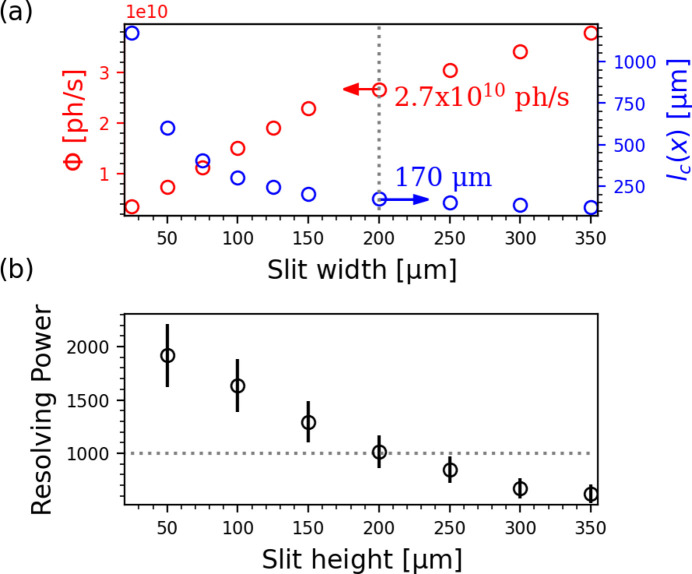
(*a*) Total photon flux (red), horizontal coherence length (blue), and (*b*) resolving power at the mask plane of beamline branch B for different SSA sizes. The resolving power is shown as a function of SSA height because the energy resolution of the PGM is defined only by the vertical SSA size. The grey dotted lines correspond to the maximum SSA size that provides coherent illumination of a representative grating mask (see Fig. 7 ▸).

The coherence length *l*_c_(*x*, *y*) can then be determined as the distance from the central point at which γ < 0.8. Repeated propagations were undertaken for different horizontal SSA sizes, ranging from 25 µm to 350 µm. The results of the simulations are shown in Fig. 3 ▸, which shows that the total flux at the mask plane increases linearly with horizontal SSA size from 2.7 × 10^9^ photon s^−1^ to 2.9 × 10^10^ photons s^−1^, while the horizontal coherence length at the mask plane decreases inversely proportional to the slit width, from ∼1.17 mm to ∼125 µm. Previous unpublished measurements by the authors using a Young’s double-slit array with a maximum slit separation of 30 µm showed no significant loss of visibility with any horizontal SSA size, indicating that the horizontal coherence length at 6.7 nm wavelength is always greater than 30 µm. The finite horizontal coherence may impose a practical limit on the horizontal extent of an IL grating mask as it will generally be smaller than the vertical coherence length due to the asymmetric source emittance (Table 1 ▸).

The bandwidth was measured using X-ray photoelectron spectroscopy (XPS) of the Au Fermi edge. The Fermi edge broadening was measured with different vertical SSA sizes and the energy bandwidth of the fundamental undulator harmonic at λ = 6.7 nm was estimated for each SSA size. The results are shown in Fig. 3 ▸.

## 4. Experimental measurements

**Figure 4:**
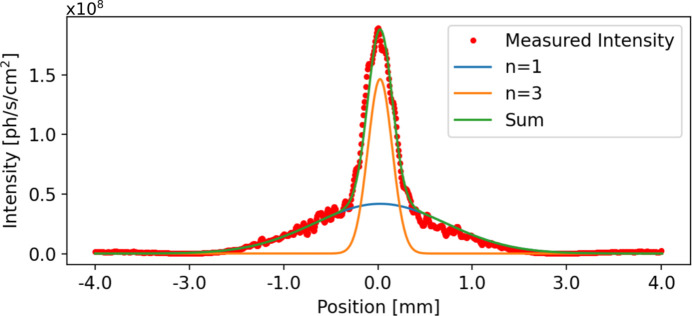
The measured horizontal intensity profile, 50 cm from the BDA with a fundamental photon energy of 185 eV and *C*_ff_ = 2. The horizontal line profile through the beam, overlaid with a fitting of two Gaussians representing the fundamental (harmonic *n* = 1) and the third harmonic (*n* = 3).

**Figure 5:**
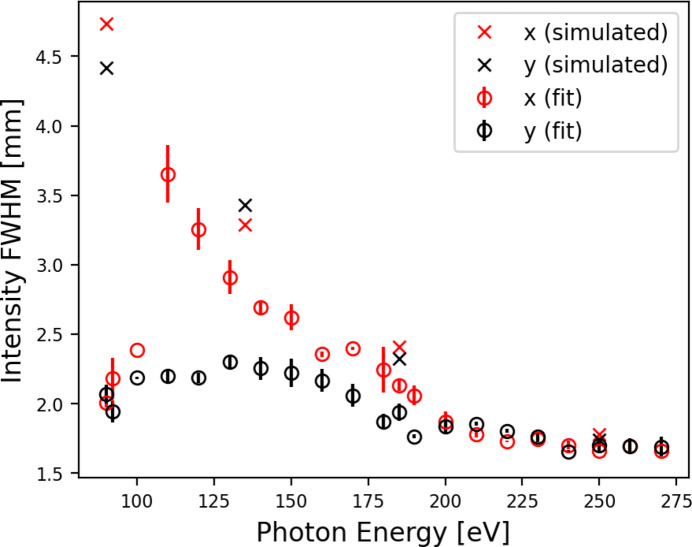
The intensity FWHM measured in the horizontal (red) and vertical (black) direction for photon energies from 90 eV to 270 eV and *C*_ff_ = 1.4. The calculated FWHM of intensity profiles generated through partially coherent wavefront propagation through the beamline model are also shown for photon energies of 90.44 eV, 135 eV, 184.76 eV and 250 eV.

Measurements of the intensity profile of the beam were taken at branch B of the beamline (Fig. 2 ▸), using an AXIS-SXR sCMOS detector with an EUV-Enhanced GSENSE400 BSI sensor (Harada *et al.*, 2019 ▸). The detector was situated *z* = 0.5 m from the beam-defining aperture (BDA) plane. At the BDA plane, a 4.064 µm-thick Ultralene (C_3_H_6_) filter was positioned in the beam path to attenuate the beam and avoid over-saturating the detector. For each measurement, the WBS were set to 0.84 mm × 1.0 mm (*x*, *y*), and the SSA was set to 25 µm × 25 µm (*x*, *y*). The PGM fixed focus constant (*C*_ff_) was set to 1.4 instead of the standard operating value of 2, as the higher harmonic content (ζ) is reduced for small *C*_ff_ values (Kleemann *et al.*, 1997 ▸); all other parameters were as shown in Table 3 ▸. These small slit sizes mean all results presented in this section show less flux and intensity than can be expected with beamline parameters optimized for high flux. The photon energy was varied from 90 eV to 270 eV in 10 eV steps, with extra steps at 92 eV (13.5 nm) and 185 eV (6.7 nm). One hundred exposures were taken at each photon energy which were processed in sets of ten. Horizontal and vertical profiles across the summed intensity in each set were fitted with two Gaussians, as shown in Fig. 4 ▸, which represent the fundamental (harmonic *n* = 1) intensity profile and the intensity profile of the third (*n* = 3) harmonic. For the fitted Gaussian representing the fundamental, the FWHM was calculated (Fig. 5 ▸).

The total photon flux of each harmonic, Φ_*n*_, was also calculated, as well as the intensity over a four-grating mask area, 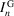 . The four-grating mask, typical of those used to produce two-dimensional patterns by IL (Mojarad *et al.*, 2015*b* ▸, 2021 ▸), consists of 50 µm × 50 µm gratings, arranged radially around the beam centre, with a 50 µm × 50 µm central beam stop. A schematic of the grating mask area over the two-dimensional beam intensity is shown later in Fig. 7. Errors for each fitted parameter have been taken as the standard deviation of the fitted value for each set of ten processed images. For each experimentally measured beam parameter discussed henceforth, equivalent parameters extracted from the partially coherent simulated intensity at 90.44, 135, 184.76 and 250 eV have been included for comparison.

The total flux was measured again as a function of photon energy using the AXUV100 photodiode at branch A (Fig. 2 ▸). Photodiode measurements were adjusted to account for the reflectivity of the extra mirror used in branch A. Photodiode measurements were taken with an SSA size of 14000 µm horizontal (*i.e.* wide open) × 20 µm vertical (branch A), while intensity measurements were taken with an SSA of 25 µm × 25 µm (branch B). The difference in horizontal SSA size between branch A and branch B leads to a 100-fold increase in flux at the photodiode, which has been accounted for by scaling the photodiode flux values in Fig. 7 accordingly.

Measurements of the harmonic content of the beam, ζ, were taken using XPS of Au 4*f*_7/2_ peaks corresponding to different undulator harmonics. Through analysis of the relative peak areas, an estimate of ζ was calculated for fundamental photon energies from 130 eV to 350 eV in 20 eV steps, 

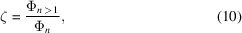

where Φ_*n*>1_ is the photon flux contained in higher undulator harmonics and 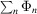 is the total flux.

## 5. Experimental results

### 5.1. Beam profile

The FWHM of the fundamental (*n* = 1) intensity obtained from the fit to the measured intensity distribution at the grating mask plane is shown in Fig. 5 ▸ for photon energies 90 eV to 270 eV. Comparison with simulated intensity FWHM shows a similar dependence on photon energy. However, at low photon energies the comparison between the two methods shows less agreement, which may be attributed to uncertainties resulting primarily from the low signal-to-noise for the intensity measurements taken with low photon energy.

### 5.2. Higher-harmonic content

**Figure 6:**
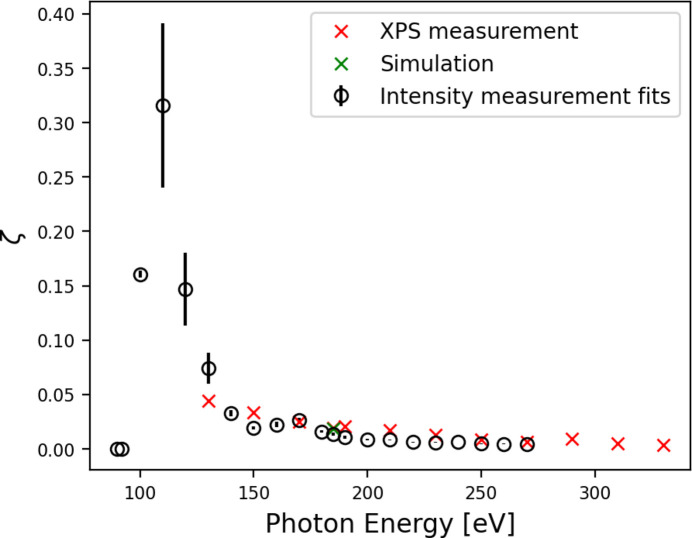
Higher harmonic content (ζ) measurements using direct beam measurements (black) and XPS measurements (red). A single calculation from simulation has been included at 184.76 eV (green).

Measurements were taken by XPS with *C*_ff_ values of 2 and 1.4. The total ζ was found to significantly reduce at *C*_ff_ = 1.4 for all photon energies measured as expected (Kleemann *et al.*, 1997 ▸), with a reduction from ∼10% harmonic content at *C*_ff_ = 2 to ∼2% at *C*_ff_ = 1.4 for a fundamental photon energy of 185 eV. The results of the harmonic content measurements are shown in Fig. 6 ▸ for *C*_ff_ = 1.4 and compared with the harmonic contamination given by the fitting of Gaussians to the direct intensity measurements. The XPS measurements show good agreement with the direct beam measurements when comparing total intensity, and the simulation results show close agreement to both measurements.

### 5.3. Flux and intensity at the mask plane

**Figure 7:**
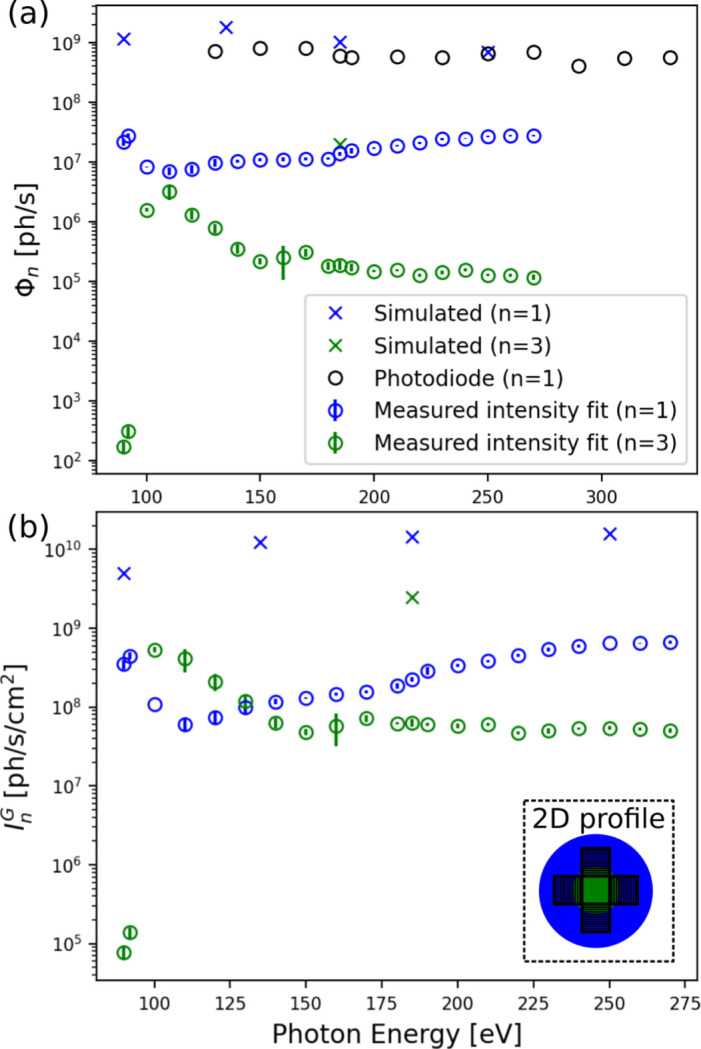
(*a)* The simulated and measured total flux (Φ_*n*_) and (*b*) the total intensity over the area of a four-grating mask ( 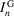 ) consisting of 50 µm × 50 µm gratings arranged symmetrically with a 50 µm gap in the centre (shown in inset, where the gratings are shown as black squares, the *n* = 1 harmonic in blue, and the *n* = 3 harmonic in green). Flux and intensity measurements were taken at branch B, while flux measurements were also taken on branch A using an AXUV100 photodiode. *C*_ff_ = 1.4 for all measurements shown.

The total flux as a function of photon energy is shown in Fig. 7 ▸ as measured by three different methods: the AXUV100 photodiode at branch A beamline (Fig. 2 ▸), the fundamental fitting of the direct beam intensity measurements taken at branch B, and simulated wavefront propagation.

The intensity measured over a typical IL-grating area, 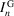 , described earlier and shown in Fig. 7 ▸, is also included for each photon energy.

The simulated results show significantly greater flux and intensity over the grating area compared with the direct intensity measurements; however, they show close agreement with the flux measurements using the photodiode. Fig. 7 ▸ also shows that the intensity-on-mask of *n* = 1 at 185 eV is 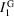 ≃ 2 × 10^8^ photons s^−1^ cm^−2^, which gives a dose-on-mask of just *D*_m_ ≃ 6 × 10^−6^ mJ s^−1^ cm^−2^. However, recall that the intensity measurements shown in Fig. 7 ▸ were made with ‘low flux’ beamline settings, using small WBS and SSA sizes to avoid over-saturation of the detector. Using ‘high flux’ settings, the intensity at the mask plane is expected to be closer to the simulated results shown in Fig. 3 ▸. Hence, the results shown in Fig. 7 ▸ serve mainly to confirm that the simulation can accurately predict intensity at the mask plane.

To evaluate the beamline’s suitability for IL, partially coherent wavefront propagation was used to calculate Φ_1_ and 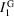 as a function of SSA size for a photon energy of 185 eV. The SSA height and width were kept equal and varied from 200 µm to 1500 µm in 100 µm steps. 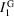 was found to reach a maximum of 5.85 × 10^13^ photons s^−1^ cm^−2^ for a 300 µm × 300 µm SSA. For the same representative grating with efficiency η = 6% illustrated in Fig. 7 ▸, this value of 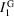 corresponds to a value of *D*_w_ equal to 5.46 mJ s^−1^ cm^−2^. For *D*_m_ = 11 mJ cm^−2^, the minimum exposure time is then 1.9 s. The coherence length at the mask plane for these settings is 137 µm, which would allow for the coherent illumination of a mask containing 45 µm gratings.

## 6. Conclusion

Partially coherent wavefront propagation simulations have been used to accurately model the SXR beamline at the Australian Synchrotron. The model was shown to give a quantitative representation of photon flux that agrees with direct experimental measurements using an AXUV100 photodiode. However, the simulated flux and the photodiode flux measurements did not show agreement with the direct intensity measurements. This could be due to an error in the thickness and density of the Ultralene filter used to attenuate the beam, which has been found to vary in thickness by 10% from the nominal value of 4 µm (Surowka *et al.*, 2020 ▸). The discrepancy could also have come from errors in simulation due to the assumption of ideal beamline elements, reflectivities and diffraction efficiencies, or a possible misalignment of the SSA in branch B of the SXR beamline, causing a drop in flux at the detector plane.

A method for higher harmonic suppression by adjusting *C*_ff_ of the PGM from 2 to 1.4 was found to reduce total harmonic contamination at the mask plane from ∼10% to ∼2%.

The beamline was shown to possess adequate power and coherence for IL at EUV/SXR wavelengths when operating with a WBS of 4 mm × 3 mm and an SSA of 300 µm × 300 µm, allowing for 1.9 s patterning of a 45 µm maximum grating size, which is compatible with that used at other EUV-IL facilities (Sahoo *et al.*, 2023 ▸). The exposure time is sufficiently short for maintaining the nanometre mechanical stability required for studying lithographic processes at high spatial resolution. Greater power is possible at the cost of coherence — and therefore grating size — with experimental evidence that the transverse coherence length exceeds 30 µm for any choice of SSA size.

## Figures and Tables

**Table 1:** Key values for the Australian Synchrotron storage ring; parameters obtained from Wootton *et al.* (2012 ▸)

	Parameter	Value
*E*_0_	Beam energy	3.01 GeV
*I*_0_	Current	0.2 A
σ_*E*_	RMS energy spread	0.1021%
ε_*x*_	Horizontal emittance	10 nm rad
ε_*y*_	Vertical emittance	0.009 nm rad
β_*x*_	Horizontal beta function	9 m
β_*y*_	Vertical beta function	3 m

**Table 2:** Key values for the APPLE-II undulator used for the SXR beamline at the Australian Synchrotron *K*_u_, which determines *B*_u_, was adjusted to produce a fundamental harmonic at 184.76 eV. Other values obtained from Wootton *et al.* (2012 ▸).

	Parameter	Value
*L*_u_	Undulator length	1.875 m
λ_u_	Undulator period length	75 mm
*N*_u_	Number of undulator periods	25
*B*_u_	Peak magnetic field	0.4611 T
*K*_u_	Deflection parameter	3.230

**Table 3:** Typical geometry of the optical elements used in the model of the beamline and IL optics The defining characteristics of a mirror are given as the major radius, *R*, and minor radius, ρ. The defining characteristic of a grating is the periodicity, and of a mask is the grating size, *G*. Aperture sizes shown were used for all simulations and measurements shown in this work unless stated otherwise.

Element name	Element type	Propagation distance (m)	Distance from source (m)	Dimensions (mm)	Defining characteristics	Incident angle (°)
White-beam slits	Aperture	14.30	14.30	4 × 3	N/A	90
Toroidal mirror	Mirror	0.82	15.12	420 × 30	*R* = 6668 m, ρ = 5.262 m	1
Planar mirror	Mirror	3.38	18.50	460 × 50	N/A	2
Grating	Grating	0.20	18.70	150 × 20	250 lines mm^−1^	1.173
Exit aperture	Aperture	1.00	19.70	10 × 20	N/A	90
Cylindrical mirror	Mirror	0.60	20.30	240 × 40	*R* = 100 km, ρ = 0.2443 m	1.5
Secondary source aperture	Aperture	6.50	26.80	0.025 × 0.025	N/A	90
Grating mask	Mask	9.50	36.30	0.15 × 0.15	*G* = 50 µm	90
